# Adaptation Mechanism of Roots to Low and High Nitrogen Revealed by Proteomic Analysis

**DOI:** 10.1186/s12284-020-00443-y

**Published:** 2021-01-07

**Authors:** Wei Xin, Lina Zhang, Jiping Gao, Wenzhong Zhang, Jun Yi, Xiaoxi Zhen, Congyuan Bi, Dawei He, Shiming Liu, Xinyu Zhao

**Affiliations:** 1 Key Laboratory of Northern Japonica Rice Genetics and Breeding, Ministry of Education and Liaoning Province, Key Laboratory of Northeast Rice Biology and Genetics and Breeding, Ministry of Agriculture, Rice Research Institute of Shenyang Agricultural University, Shenyang, 110866 China; 2 Graduate School of Agricultural Science, Tohoku University, Sendai, 981-8555 Japan

**Keywords:** Nitrogen, Proteomics, Root system morphology, Rice

## Abstract

**Background:**

Nitrogen-based nutrients are the main factors affecting rice growth and development. Root systems play an important role in helping plants to obtain nutrients from the soil. Root morphology and physiology are often closely related to above-ground plant organs performance. Therefore, it is important to understand the regulatory effects of nitrogen (N) on rice root growth to improve nitrogen use efficiency.

**Results:**

In this study, changes in the rice root traits under low N (13.33 ppm), normal N (40 ppm) and high N (120 ppm) conditions were performed through root morphology analysis. These results show that, compared with normal N conditions, root growth is promoted under low N conditions, and inhibited under high N conditions. To understand the molecular mechanism underlying the rice root response to low and high N conditions, comparative proteomics analysis was performed using a tandem mass tag (TMT)-based approach, and differentially abundant proteins (DAPs) were further characterized. Compared with normal N conditions, a total of 291 and 211 DAPs were identified under low and high N conditions, respectively. The abundance of proteins involved in cell differentiation, cell wall modification, phenylpropanoid biosynthesis, and protein synthesis was differentially altered, which was an important reason for changes in root morphology. Furthermore, although both low and high N can cause nitrogen stress, rice roots revealed obvious differences in adaptation to low and high N.

**Conclusions:**

These results provide insights into global changes in the response of rice roots to nitrogen availability and may facilitate the development of rice cultivars with high nitrogen use efficiency through root-based genetic improvements.

**Supplementary Information:**

The online version contains supplementary material available at 10.1186/s12284-020-00443-y.

## Background

Rice is one of the most important food crops worldwide, and sustainable development of rice agriculture is an essential part to ensure global food security (Hua et al. 2015). With the acceleration of urbanization, the area of arable land is shrinking, and food production security must be achieved by increasing yields (Sun et al. 2014). In recent years, researchers have steadily increased rice yield through measures such as improving rice varieties, developing advanced cultivation techniques, and increasing production inputs. Among them, increasing the nitrogen input is one of the most effective measures to increase rice production (Godfray et al. 2010; Liu et al. 2013). However, given the significant increase in rice production, excessive and inappropriate nitrogen fertilizer input has also led to a series of problems, such as a decrease in the nitrogen fertilizer utilization rate and an increase in production cost, air pollution, and water pollution cost (Hakeem et al. 2011; Gutiérrez 2012).

Roots not only constitute important organs of rice that absorbs nutrients and moisture but are also the site for assimilation, transformation and synthesis of several substances, such as phytohormones, organic acids, etc. (Wu and Cheng 2014; Meng et al. 2019). Therefore, the morphology and physiology of rice roots are closely related to nitrogen uptake and utilization (Zhang et al. 2009; Lynch 2013). Ju et al. (2015) suggested that N-efficient rice varieties have larger root biomass, deeper root distribution, longer root length, and greater root length density. At the same time, changes in nitrogen availability affect root morphology and physiology (Xu et al. 2012). The morphological and physiological responses of rice roots to changes in N availability have been studied intensively (Marschner 1995; Mi et al. 2010; Ju et al. 2015). Mild nitrogen deficiency can promote root growth, which is conducive to deep root penetration; appropriate nitrogen supply can increase root number, biomass and density; but excessive nitrogen supply can inhibit root growth (Walch-Liu 2006; Francisco et al. 2015). Ju et al. (2015) showed that within a certain range, root biomass and root oxidation activity increased with increasing nitrogen levels. However, the molecular regulatory mechanisms underlying the morphological and physiological acclimation to different nitrogen levels remain largely unknown. Understanding these mechanisms is of great importance for not only the scientific community involved in research on rice biology but also practical rice breeders, as knowledge of rice root responses to nitrogen availability enables the breeding of rice with high nitrogen use efficiency.

In recent years, the development and application of high-resolution mass spectrometry (MS) and information processing technologies have advanced. Comparative proteomic analysis plays an important role in understanding the mechanisms by which plants adapt to biotic and abiotic stress (Turek et al. 2015; Wang et al. 2016; Hao et al. 2017; Tian et al. 2018a; Tian et al. 2018b). However, to the best of our knowledge, almost no research has been conducted on the response of rice roots to nitrogen nutrition through integrated morphological and proteomic analysis.

In this study, rice plants were subjected to low, control and high N treatments. The changes in root traits and root proteome profiles were analyzed after 30 days. We found that compared with normal N conditions, root biomass and the total root length increased under low N conditions and decreased under high N conditions. Differences in protein abundance involved in cell division and expansion, lignin synthesis, and nascent protein synthesis could be important factors leading to root morphological changes. The objective of this research is to provide the fundamental information needed to identify strategies employed by rice roots to cope with low and high N stress. This information could be used for research focused on improving nitrogen use efficiency and production of rice.

## Results

### Architectural Responses of Rice Roots to Nitrogen Availability

**Fig. 1:**
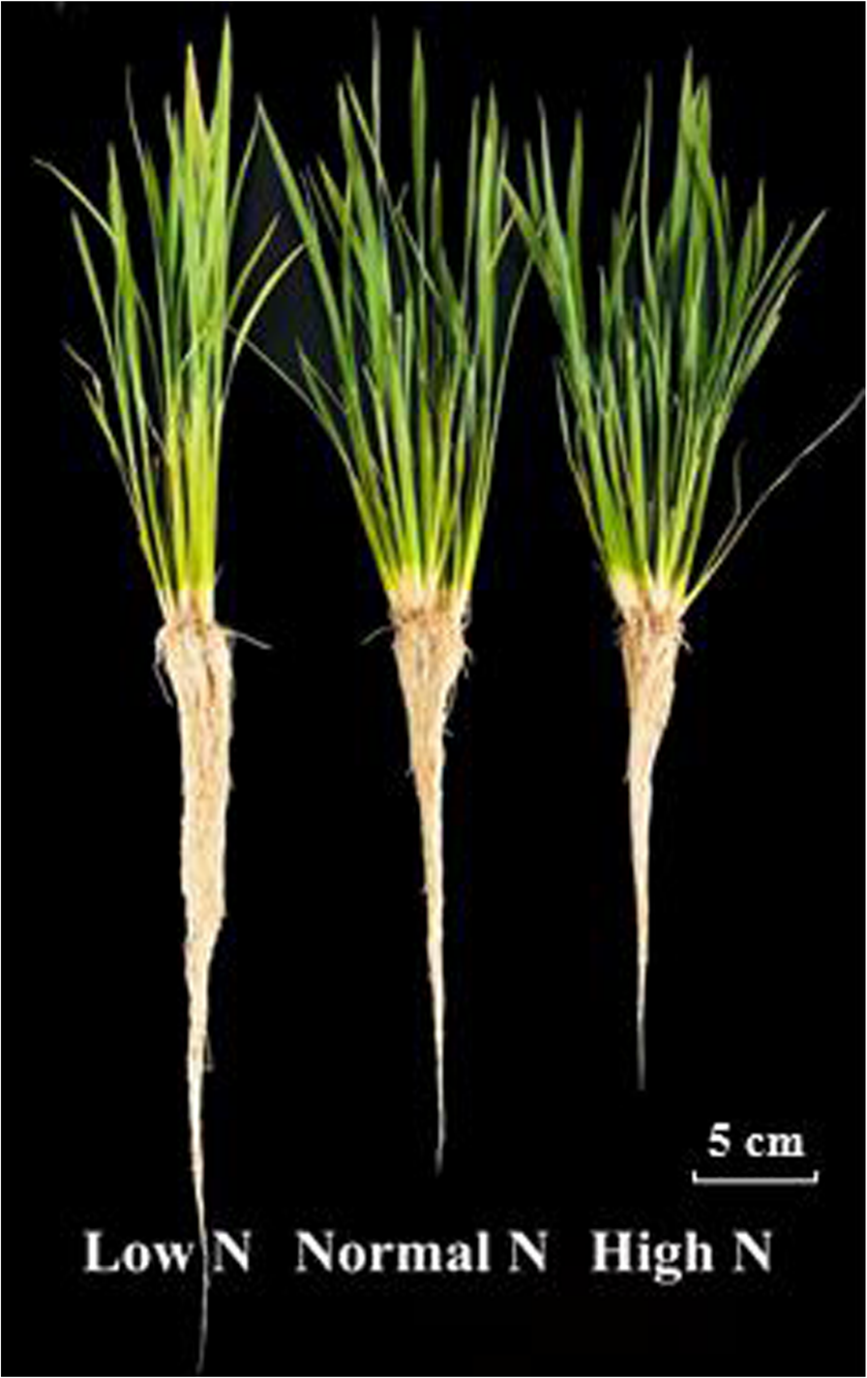
Effects of N treatments on roots morphology

To understand the response mechanism of rice roots to low and high nitrogen, the current study conducted an integrated morphological and proteomic analysis. As shown in Table 1, root biomass (Xin et al. 2019b) and total root length significantly decreased under high N conditions as compared to normal N conditions (Table 1, Fig. 1), and increased under low N conditions. The maximum root length significantly increased under low N conditions, but no significant effect was observed under high N conditions. The root diameter was not affected under low N conditions, but it significantly decreased under high N conditions. Low N led to a considerable reduction in root number, but no change under high N. As we previously reported, the nitrogen content increased with increasing nitrogen supply levels (Xin et al. 2019b).

**Table 1:** Effects of nitrogen availability on root morphology and nitrogen content

Treatment	Low N	Normal N	High N	***P*** value
Root biomass (g)	1.07 ± 0.08a	0.84 ± 0.03b	0.66 ± 0.03c	***
Total root length (cm)	8032.50 ± 396.01a	6694.83 ± 150.31b	5423.52 ± 300.58c	***
Maximum root length (cm)	31.30 ± 0.40a	27.96 ± 0.62b	27.41 ± 1.08b	**
Root diameter (mm)	0.46 ± 0.02a	0.45 ± 0.03a	0.36 ± 0.04b	*
Root number	181.67 ± 4.93b	259.33 ± 7.77a	245.00 ± 8.72a	***
Root nitrogen content	2.05 ± 0.08c	2.44 ± 0.11b	3.39 ± 0.13a	***

Values labeled with different letters in the same row indicate significant differences between the nitrogen treatments. As evaluated with ANOVA with Fisher’s LSD, *p < 0.05; **p < 0.01; ***p < 0.001, *n* = 3. The root biomass and root nitrogen content data have been reported in our previous study (Xin et al. 2019b)

**Fig. 2:**
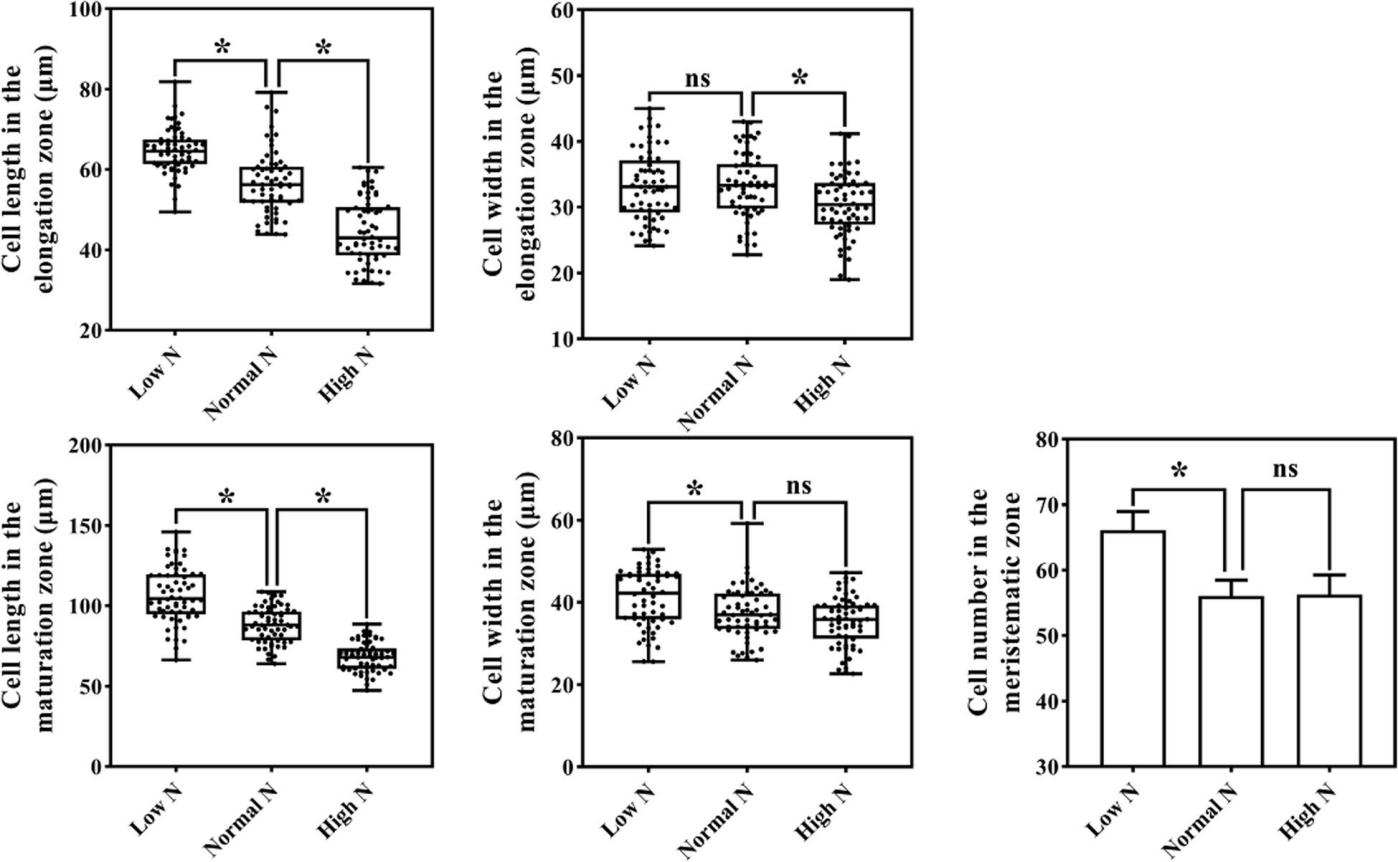
Changes of cell size in rice roots in response to low and high N. **a** cell length in the elongation zone. **b** cell width in the elongation zone. **c** cell length in the maturation zone. **d** cell width in the maturation zone. **e** cell number in the meristematic zone, 10,000 μm^2^. Asterisks indicate significant differences between the two N treatments, (as evaluated with ANOVA with Fisher’s LSD, *, *p* < 0.05, ns, not significant. *n* = 60 (cell length and width), *n* = 9 (cell number))

To determine whether the cell division and elongation were affected under different N treatments, the number and size of cells in root tips were examined. As shown in Fig. 2, compared to normal N conditions, the length/width of cell in the elongation zone revealed increased/no significant change under low N condition, while the length/width of cell in the elongation zone revealed decreased/decreased under high N condition. Compared to normal N conditions, the length/width cell in the maturation zone revealed increased/increased under low N condition, while the length/width of cell in the maturation zone revealed decreased/no significant change under high N condition. Compared to normal N conditions, cell number in the meristematic zone increased under low N conditions, but did not change significantly under high N conditions. This result indicates that changes in cell division and cell expansion may contribute to the changed root length of rice under low and high N conditions.

### Overview of Root Proteome Profiles in Response to Different N Treatments

**Fig. 3:**
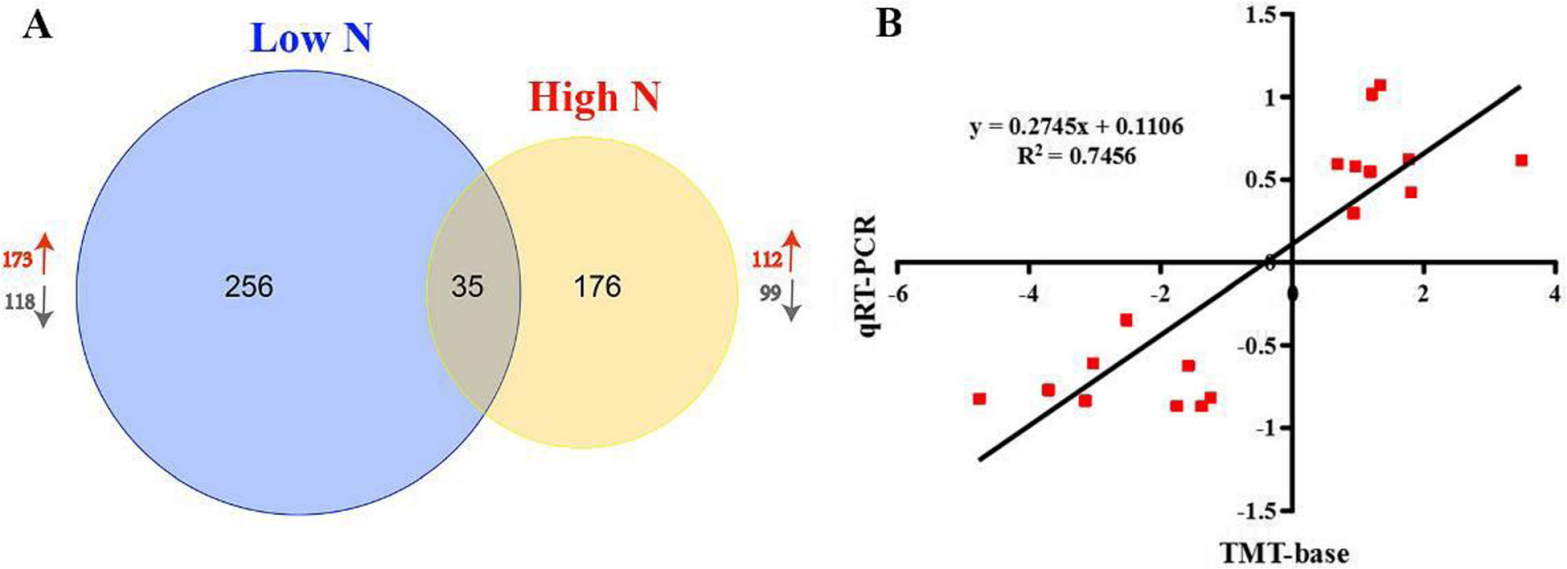
Proteomic analysis of rice roots under low and high N conditions. **a** the total number of DAPs under low and high N conditions; **b** a qRT-PCR assay was carried out for 9 randomly selected DAPs. The values are the log_2_ (Fold charge) (low N/control N or high N/control N) for genes or proteins. The correlation coefficient (*R*^2^) is indicated in the figure

To understand the molecular basis of changes in rice root morphology and function under different N regimes, rice root samples under the three N conditions (low N, normal N, and high N) were characterized with tandem mass tag (TMT)-based proteomics. A total of 6169 rice root proteins were identified under the three N conditions (Fig. S1). Compared to normal N, there were 291 differentially abundant proteins (DAPs) in the roots under low N conditions, 173 of which exhibited increased abundance, while 118 exhibited decreased abundance. Compared to normal N, there were 211 DAPs in the roots under high N conditions, 112 of which exhibited increased abundance, while 99 exhibited decreased abundance. More proteins increased in abundance than decreased in abundance under both low and high N conditions (Fig. 3a). Among them, 35 proteins were identified as DAPs under both low and high N conditions, from which, nine DAPs were selected for qRT-PCR analysis. The proteome profiles were reliable as a high correlation (0.74) was detected between the qRT-PCR and TMT quantification. (Fig. 3b).

**Fig. 4:**
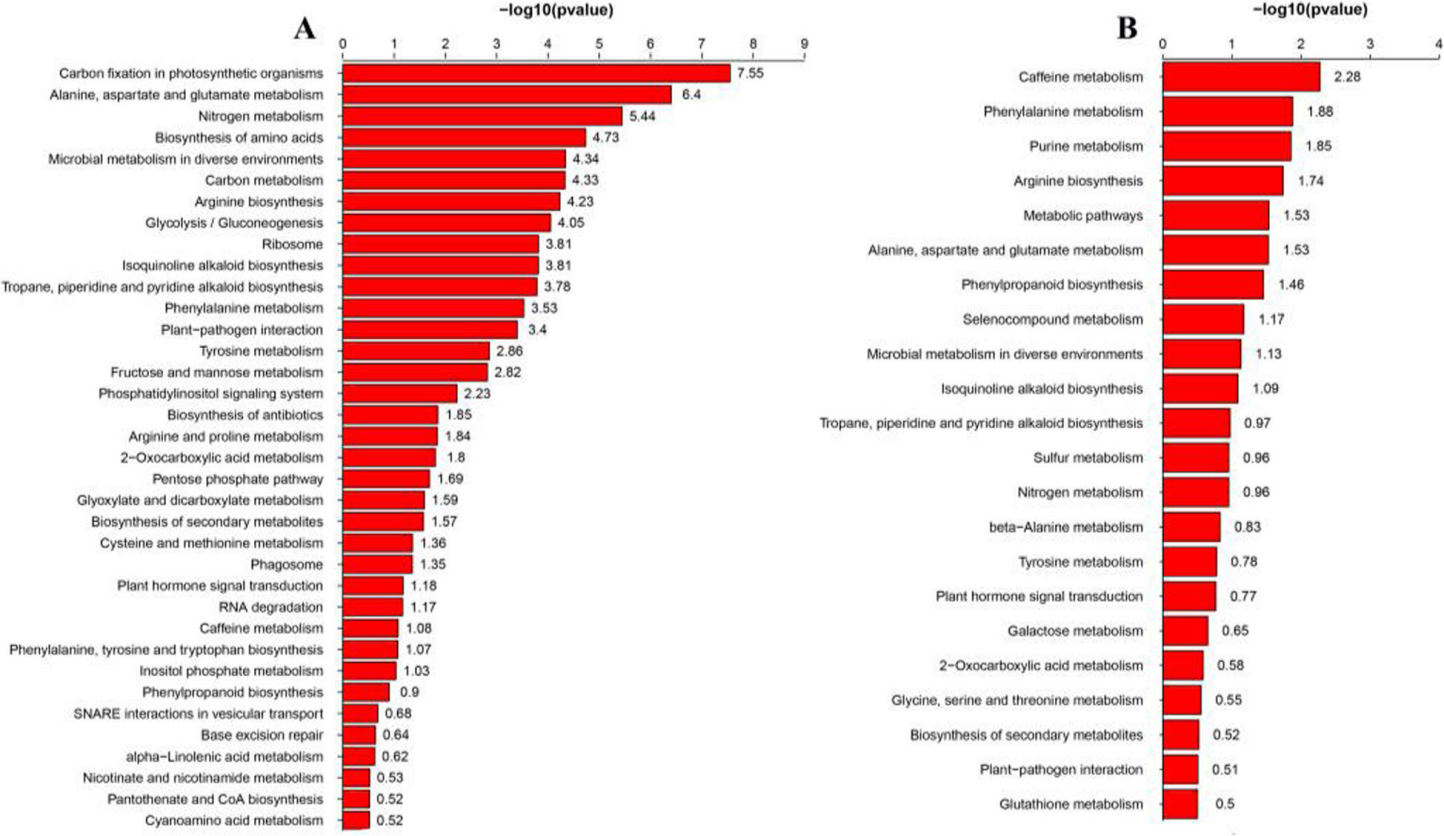
KEGG analysis of DAPs in rice roots under low and high N conditions. a, KEGG analysis of all identified DAPs under low and high N conditions; b, KEGG analysis of DAPs shared by the low and high N conditions. The -log10 values of Fisher’s exact test *p* values for the significant pathways are shown on the X axis

To obtain insights into the main biological functions of DAPs and the metabolic processes involved, Kyoto Encyclopedia of Genes and Genomes (KEGG) analyses were performed. All DAPs enrichment analyses showed that carbon metabolism, nitrogen metabolism, amino acid metabolism, stress and defense-related metabolism, and protein synthesis processes were significantly enriched (Fig. 4a). In addition, enrichment analysis of the DAPs in low and high N suggested that the top three enriched pathways were caffeine metabolism, phenylalanine metabolism, and purine metabolism (allantoin metabolism) (Fig. 4b).

### Nitrogen Uptake and Assimilation in Rice Roots

**Fig. 5:**
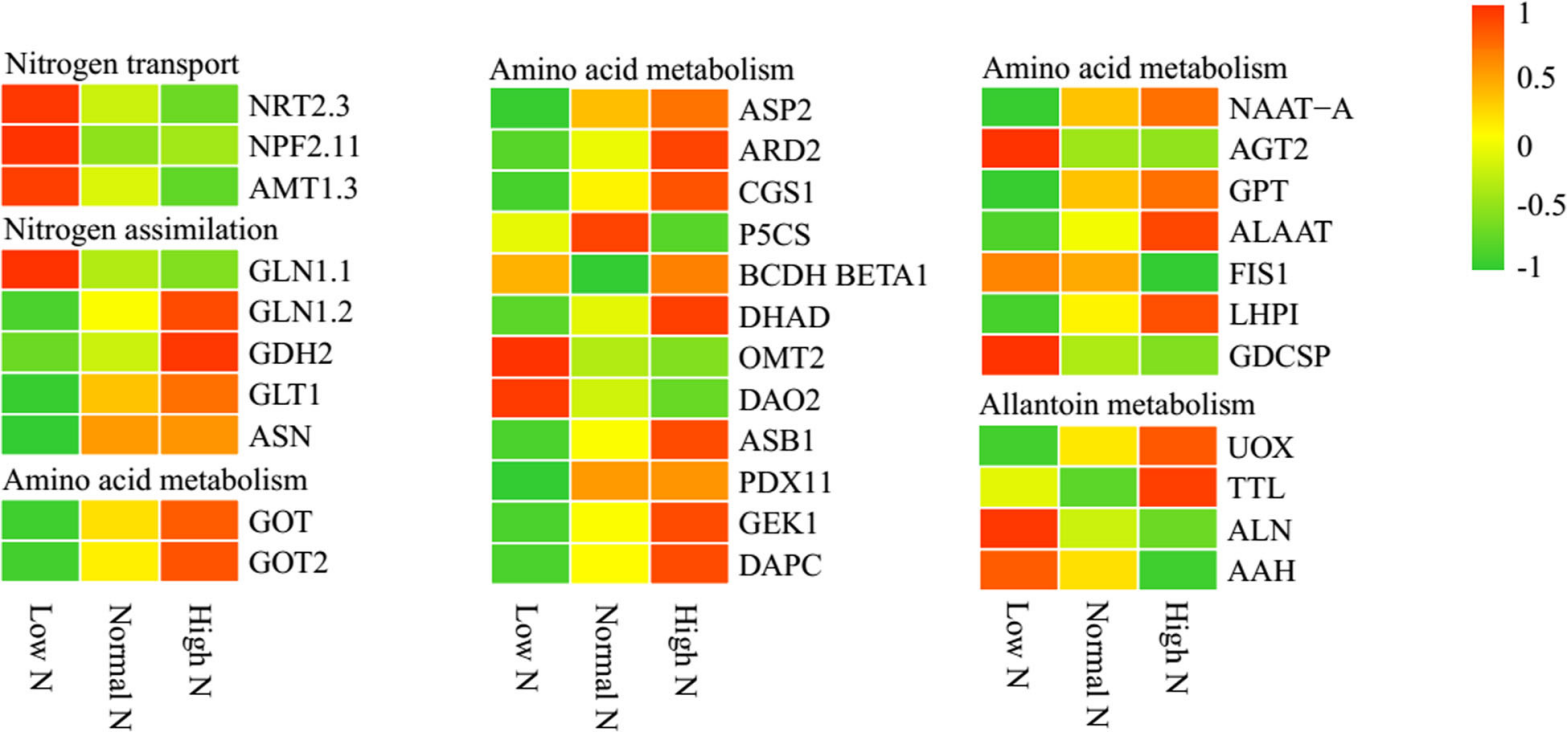
Differential changes in nitrogen metabolism-related proteins in rice roots under low and high N conditions. Proteins were displayed by different colors. Relative levels of abundance are showed by a color gradient from low (green) to high (red). For each heatmap from left to right: Low N, Normal N, High N

As shown in Fig. 5, compared with normal N conditions, the nitrogen transporter proteins NRT2.3, NPF2.11 and AMT1.3 exhibited increased abundance under low N conditions, while AMT1.3 exhibited decreased abundance under high N conditions. Five nitrogen assimilation-related proteins were identified as DAPs; all except GLN1.1 exhibited decreased abundance under low N conditions. GDH2 exhibited increased abundance under high N conditions. Nineteen amino acid metabolism-related proteins were identified as DAPs, 15 were identified under low nitrogen conditions, all except OMT2, AOC2, AGT2 and GDCSP exhibited decreased abundance. Eight were identified under high N conditions, all except P5CS and AOC2 exhibited increased abundance. Notably, allantoin metabolism-related proteins exhibited opposite changes in protein relative abundance under low and high N conditions.

### Cell Structure and Growth-Related Proteins

**Fig. 6:**
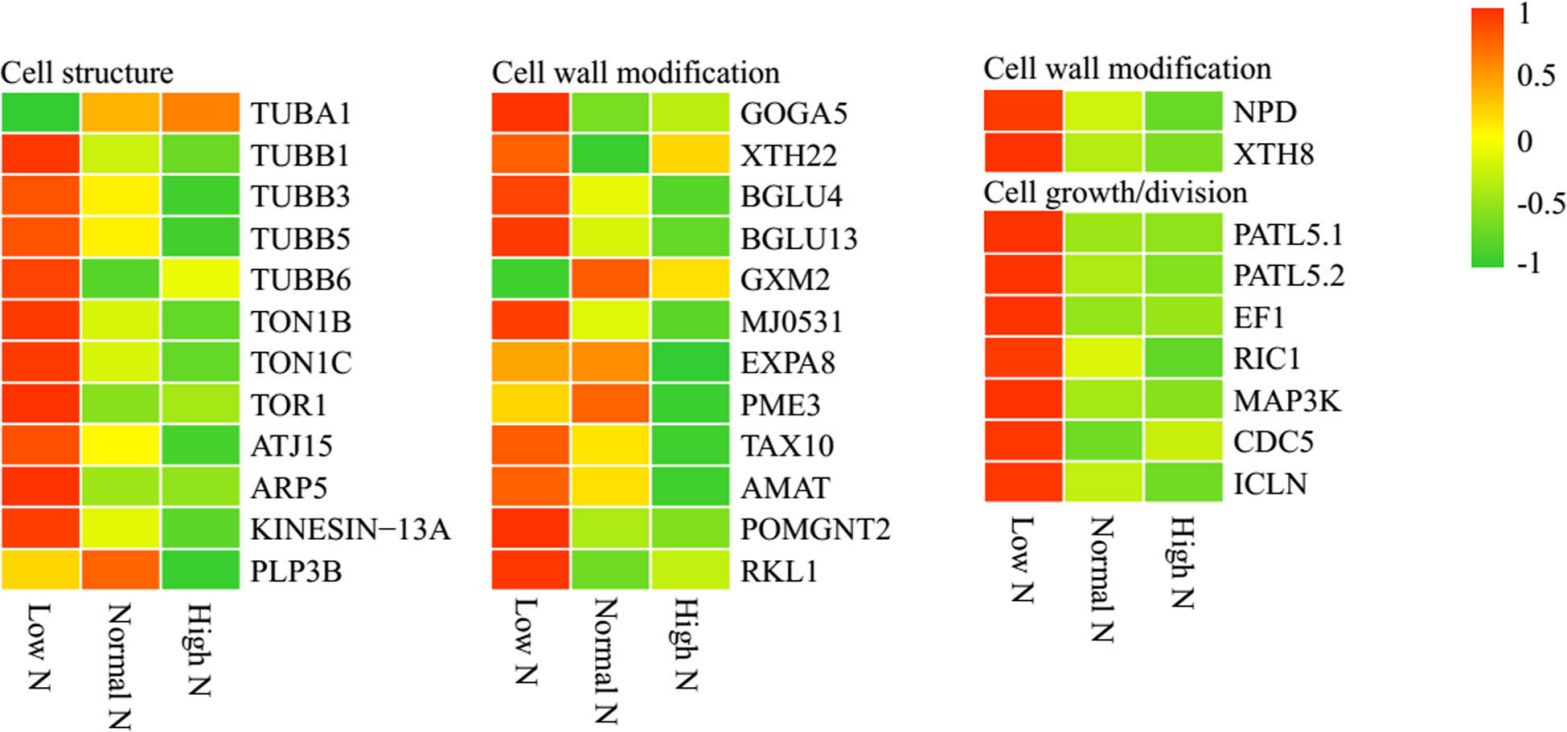
Differential changes in cell structure- and growth-related proteins in rice roots under low and high N conditions. Proteins were displayed by different colors. Relative levels of abundance are showed by a color gradient from low (green) to high (red). For each heatmap from left to right: Low N, Normal N, High N

As shown in Fig. 6, compared with normal N conditions, 33 cell structure- and growth-related proteins were identified as DAPs. Among the cell structure-associated proteins, eight were identified as DAPs under low N conditions, and all except TUBA1 exhibited increased abundance. Additionally, six were identified as DAPs under high N conditions, which exhibited decreased abundance. Among the cell wall-related proteins, 11 were identified as DAPs under low N conditions, and all except GXM2 exhibited increased abundance, six were identified as DAPs under high N conditions, which exhibited decreased abundance. Among the cell growth/division-related proteins, seven were identified as DAPs under low N conditions, which exhibited increased abundance.

### Protein Homeostasis-Related Proteins

**Fig. 7:**
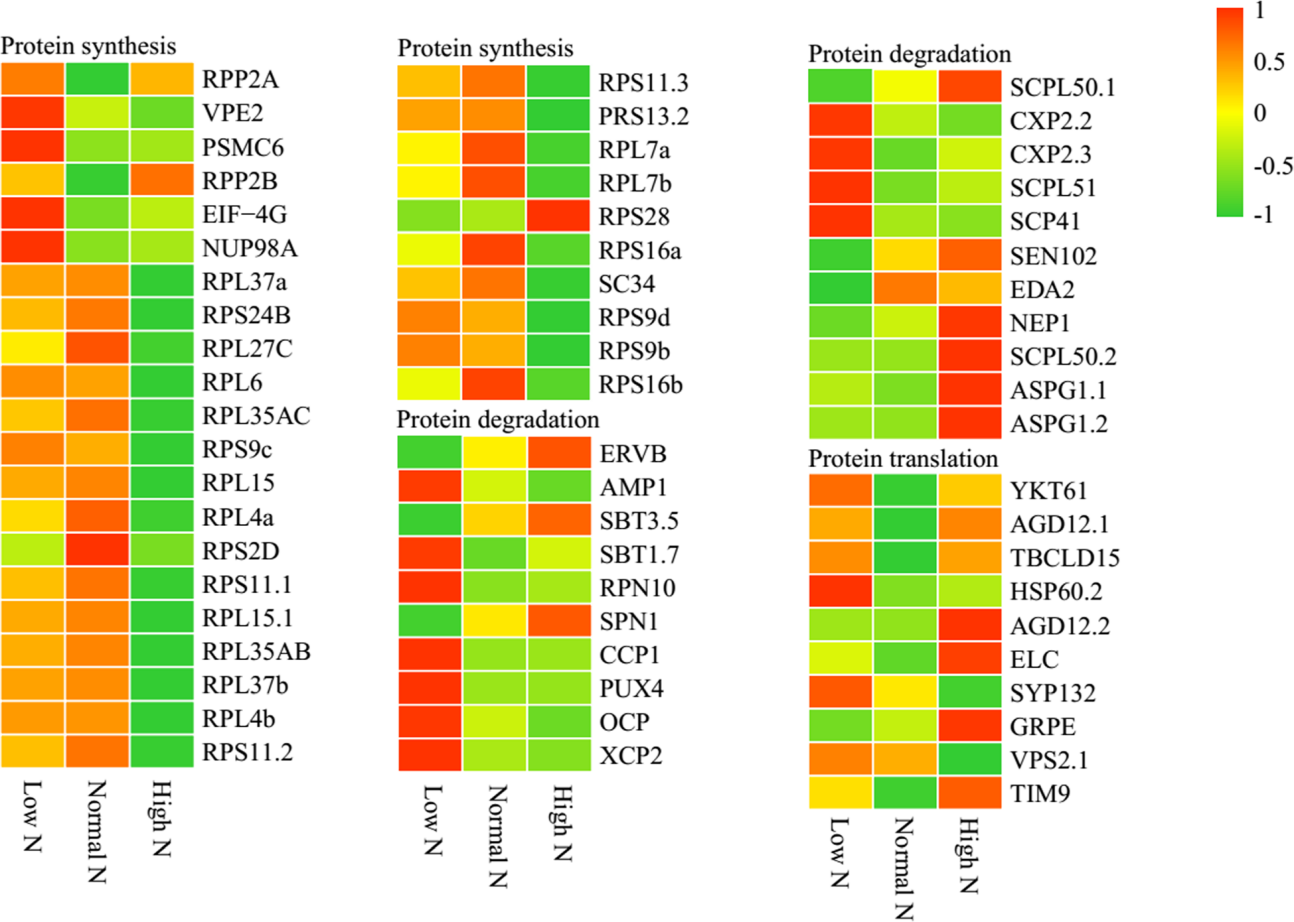
Differential changes in protein homeostasis-related proteins in rice roots under low and high N conditions. Proteins were displayed by different colors. Relative levels of abundance are showed by a color gradient from low (green) to high (red). For each heatmap from left to right: Low N, Normal N, High N

As shown in Fig. 7, compared with normal N conditions, 62 protein homeostasis-related proteins were identified as DAPs. Among the protein synthesis-related proteins, six were identified under low N conditions, which exhibited increased abundance, 25 were identified as DAPs under high N conditions, all except RPS28 showed decreased abundance. Among the protein degradation-related proteins, 18 were identified as DAPs under low N conditions, all except ERVB, SBT3.5, SPN1, SCPL50.1, SEN102, EDA2, and NEP1 exhibited increased abundance. Additionally, six were identified as DAPs under high N conditions, all except AMP1 and OCP exhibited increased abundance. Among the protein translation-related proteins, 4 were identified as DAPs under low N conditions, which exhibited increased abundance, and seven were identified as DAPs under high N conditions, all except TBCLD15, SYP132 and VPS2.1 exhibited increased abundance.

### Stress- and Defense-Related Proteins

**Fig. 8:**
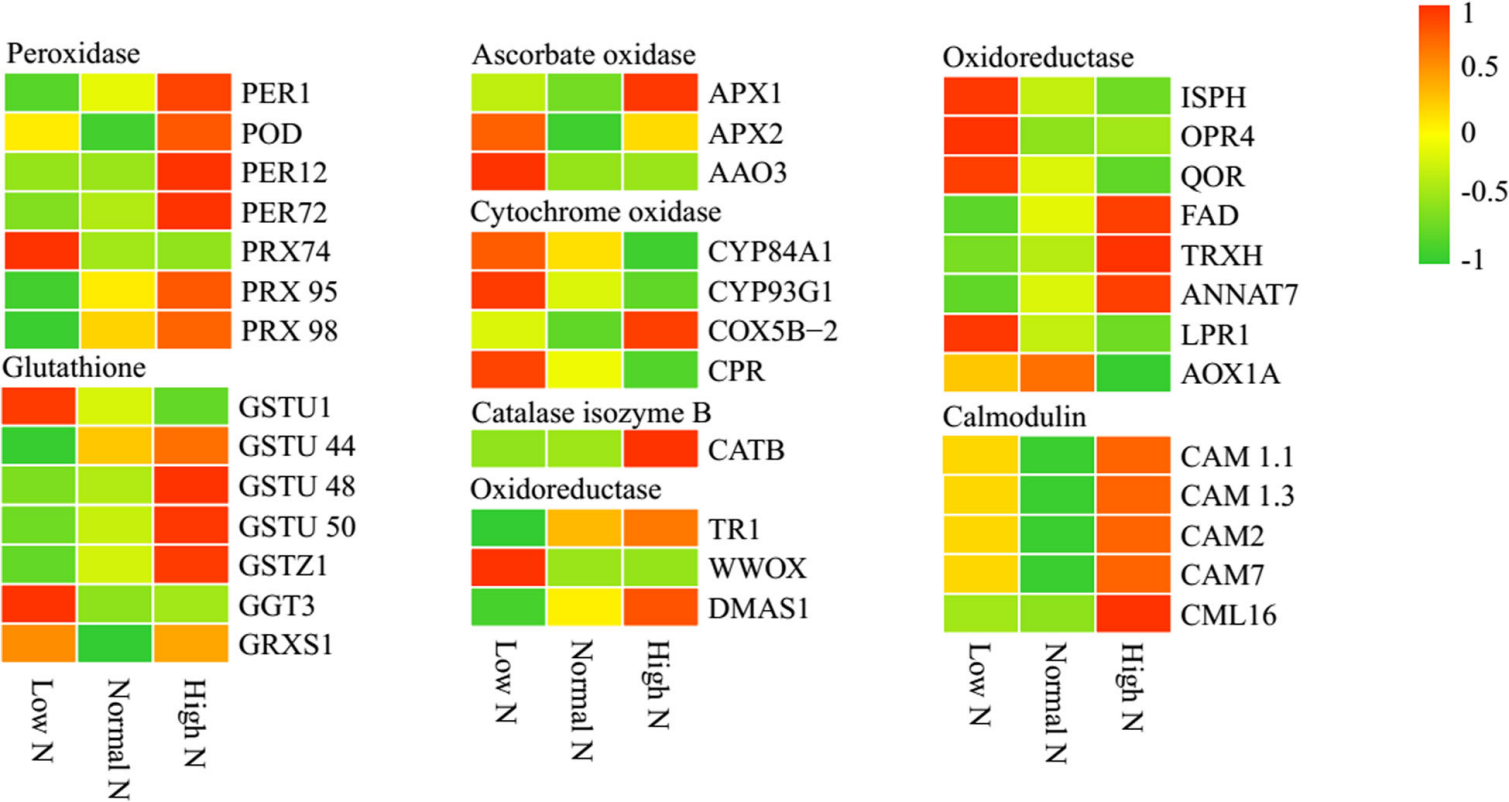
Differential changes in stress- and defense-related proteins in rice roots under low and high N conditions. Proteins were displayed by different colors. Relative levels of abundance are showed by a color gradient from low (green) to high (red). For each heatmap from left to right: Low N, Normal N, High N

As shown in Fig. 8, compared with normal N conditions, 39 stress- and defense-related proteins were identified as DAPs. Seven peroxidases were identified, four were identified under low N conditions, and all except PRX74 exhibited decreased abundance. In addition, four were identified under high N conditions, which exhibited increased abundance. Seven glutathione metabolism-related proteins were identified as DAPs, five were identified as DAPs under low N conditions, all except GSTU44 and GSTU50 exhibited increased abundance, three were identified as DAPs under high N conditions, and exhibited increased abundance. Three ascorbate-related proteins were identified as DAPs, the abundance of AAO3 and APX2 increased under low N conditions, and the abundance of APX1 increased under high N conditions. Four cytochrome oxidase-related proteins were identified as DAPs, the abundance of CPR and CYP93A2 increased under low N conditions, while the abundance of COX5B-2 increased and CYP84A1 decreased under high N conditions. The abundance of the catalase isozyme CATB increased under high N conditions. Eleven redox-related proteins were identified as DAPs, nine were identified under low N conditions, all except TR1, DMAS1, FAD and TRXH exhibited increased abundance. Four were identified as DAPs under high N conditions, all expect QOR and AOX1A exhibited increased abundance. Notably, five calmodulin-related proteins exhibited increased abundance under high N conditions.

### Phenylpropanoid Biosynthesis

**Fig. 9:**
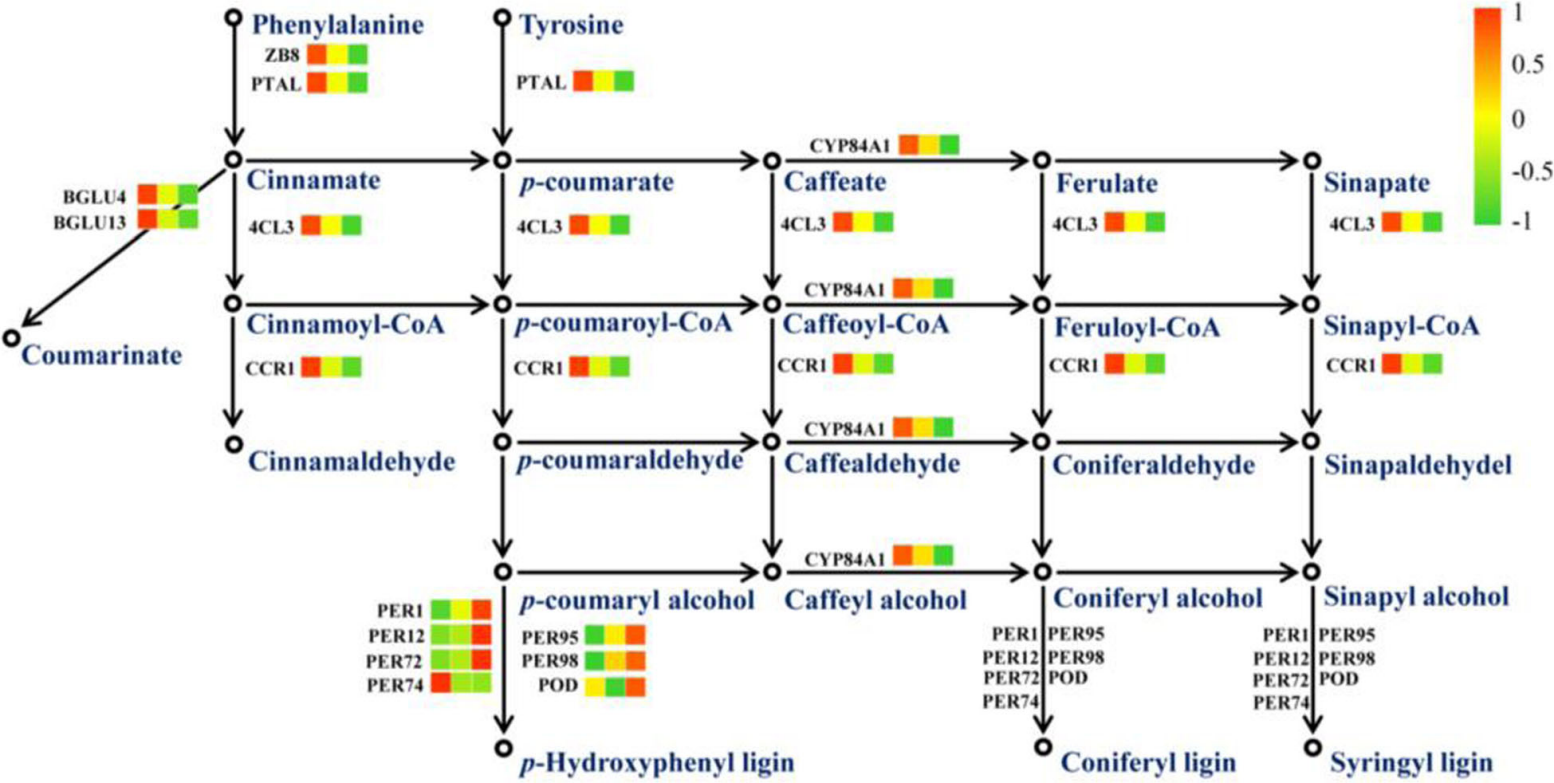
DAPs enriched in phenylpropanoid biosynthetic pathways under low and high N conditions. Proteins were displayed by different colors. Relative levels of abundance are showed by a color gradient from low (green) to high (red). For each heatmap from left to right: Low N, Normal N, High N

**Fig. 10:**
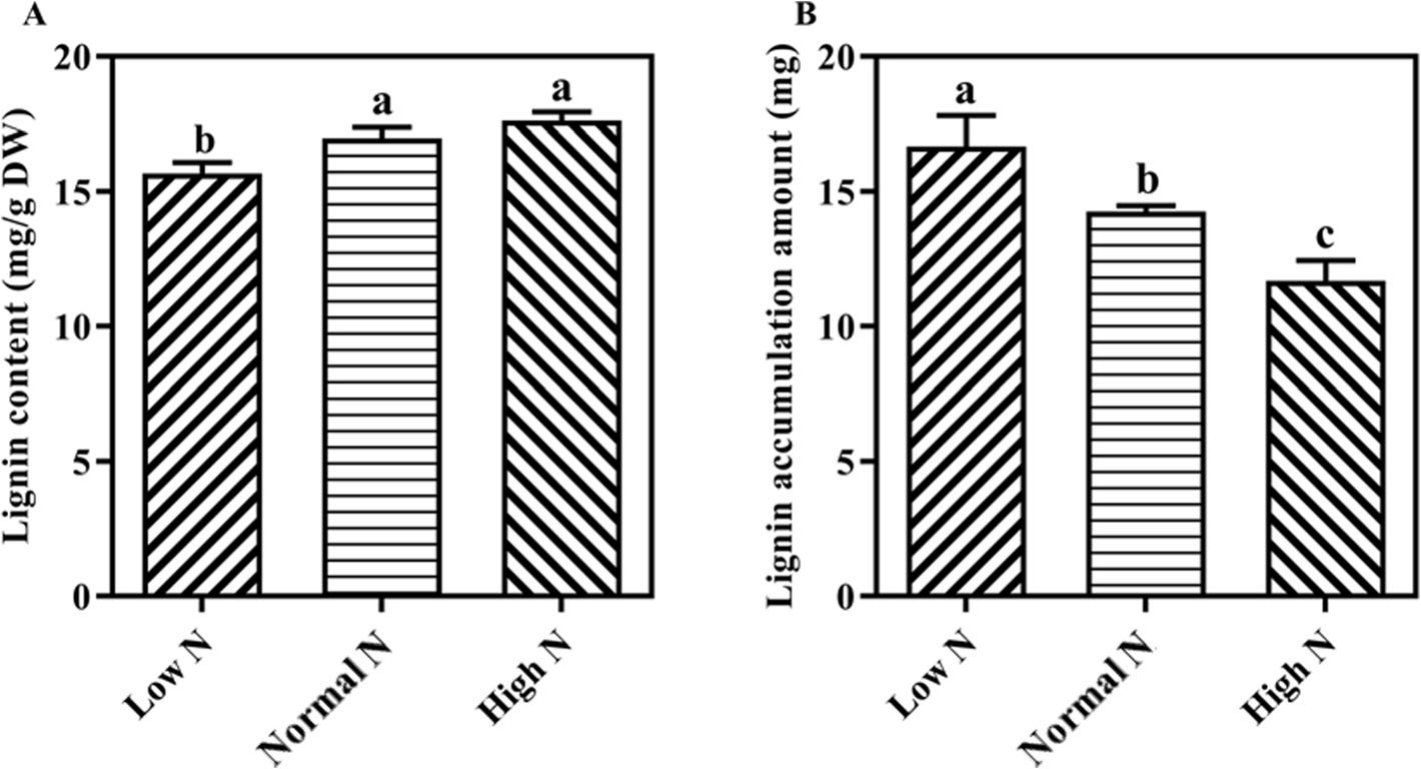
Rice root lignin content and accumulation amount response to low and high N. Values labeled with different letters in same row indicate significant difference between the N treatments, (as evaluated with ANOVA with Fisher’s LSD, *p* < 0.05, *n* = 3), DW, Dry weight

Phenylalanine metabolism and phenylpropanoid biosynthesis were enriched in both DAPs and shared DAPs (Fig. 4). As shown in Fig. 9, the abundance of the main enzymes PAL, 4CL3, CCR1 and CYP93A2 involved in phenylpropanoid biosynthesis increased under low N conditions. The abundance of the main enzymes ZB8, PAL, CCR1 and CYP84A1 involved in phenylpropanoid biosynthesis decreased under high N conditions. Root lignin content decreased under low N conditions, no significant effect was observed under high N conditions. However, root lignin accumulation amount decreased with increasing nitrogen supply levels (Fig. 10). A total of 7 peroxidases involved in phenylpropanoid biosynthesis were identified as DAPs under low and high N conditions.

## Discussion

### The Distinct and Common Adaptation of Rice Roots to Low Nitrogen and High Nitrogen

Nitrogen nutrients are the main factors affecting rice growth and development. Most previous studies focused on the effects of nitrogen deficiency (Cai et al. 2012; Yang et al. 2015; Curci et al. 2017; Subodh et al. 2018; Qin et al. 2019), while studies on the effects of N excess have been inadequate. However, the current study has shown that there were obvious differences in the morphological characteristics of rice roots under N deficiency and excess conditions, especially, root length was increased under low N conditions, while high N showed the opposite. Previous studies have shown that, when nutrients are insufficient in the environment, plants can improve nutrient acquisition by promoting root distribution in the medium, thereby maintaining survival (Saengwilai et al. 2014; Wang et al. 2019). This result indicated that root morphological characteristics are of great significance to adapt rice to nitrogen stress.

Nitrogen absorption and assimilation are interdependent in plants, and roots play a key role in this process. Inorganic nitrogen is absorbed and transported by specific transport proteins, such as ammonium transporters (AMTs) and nitrate transporters (NRTs) (Hua et al. 2015). In the present study, compared with normal N conditions, abundance of AMT1.3, NRT2.3, and NPF2.11 increased under low N conditions, while abundance of AMT1.3 decreased under high N conditions. Glutamine synthetase (GS) is a key enzyme in nitrogen assimilation (Yang et al. 2016). In rice, the GS protein family has three members, namely, GLN1.1, GLN 1.2, and GLN 1.3, and the function of GLN1.1 cannot be compensated by GLN1.2 and GLN1.3 (Tabuchi et al. 2005). In our study, we observed that compared with normal N conditions, the abundance of GLN1.1 increased under low N conditions. This result indicated that GLN1.1 may have the ability to promote nitrogen assimilation to compensate for nitrogen deficiency. In this study, compared with normal N conditions, the abundance of GDH2 increased under high N conditions, and the high abundance of GDH2 may alleviate ammonium toxicity caused by high N conditions. Interestingly, we observed different responses of allantoin metabolism to low N and high N conditions. The synthesis of allantoin was inhibited and decomposition was promoted by low N, and the opposite was observed under high N conditions. Lescano et al. (2016) showed that osmotic stress can induce the expression of UOX and AS in the allantoin synthesis pathway and inhibit the expression of ALN in the decomposition pathway, which ultimately leads to an increase in allantoin levels. We suggest that NH_3_^+^ produced by allantoin decomposition for nitrogen remobilization, may supplement N deficiency. Moreover, the accumulation of allantoin will be able to mobilize the specific response process to adapt to high N stress. Many studies have also demonstrated that plant antioxidant systems are rapidly affected by changes in nutrient conditions (Luo et al. 2015; Curci et al. 2017; Bellegarde et al. 2019). In the current study, a large number of stress-related proteins increased in abundance under low N and high N conditions. Among them, calmodulin exhibited a strong response to high N. Calmodulin is a calcium receptor that is widely found in eukaryotes and is involved in plant growth and the response to various forms of stress (Lee et al. 2005; Chinpongpanich et al. 2012).

In summary, we suggest that under low N conditions, the purpose of rice roots morphology and protein level changes is to enhance N uptake and assimilation to guarantee optimum growth. Under high N conditions, rice root may be adapted to high N stress by increasing the abundance of calmodulin and GDH2, as well as the accumulation of allantoin. Similarly, both low and high N can induce expression of antioxidant-related proteins to adapt to stress.

### Potential Regulatory Mechanisms of Root Morphology Response to Low Nitrogen and High Nitrogen

In the present study, the number and size of cells in root tips were affected by low and high N. Proteomics analysis results also shown that, the abundance of proteins involved in cell development was affected by variations in N availability, for example, the expression of microtubule-associated proteins was induced by low N but inhibited by high N. Microtubules are major structural components of eukaryotic cells and play a key role in regulating cell division, cell proliferation and cell morphology (Wasteneys and Yang 2004). Qin et al.’s (2019) research on rapeseed roots under nitrogen deficiency also found similar results. Cell elongation not only increases cytoplasm, but also promotes cell wall components (such as cellulose, hemicellulose, pectin, ect.) synthesis to maintain cell wall thickness. In the current study, proteins related to cellulose, hemicellulose, and pectin were identified as DAPs under low and high N conditions. Furthermore, compared with normal N conditions, abundance of several cell growth/division- related proteins increased under low N conditions. Therefore, the response of rice root morphology to N availability could be achieved through coordination of these relevant proteins involved in cell structure, cell wall modification and cell division. This coordination could ultimately trigger changes in root morphology with nitrogen availability. Lignin is a major component in the formation of plant skeletons and plays an important role in plant resistance to biotic and abiotic stresses. Phenylalanine/tyrosine ammonia-lyase (PTAL), 4-coumarate-CoA ligase (4CL), and cinnamoyl-CoA reductase (CCR) are key enzymes involved in the lignin synthesis pathway. In this study, we observed that the abundance of PTAL, 4CL, and CCR were induced by low N and inhibited by high N. The differences in the abundance of these proteins were in line with the changed lignin accumulation amount under low N and high N conditions. Additionally, this study suggests that the main reason for the low lignin content under low nitrogen conditions is fast root growth. Well known, biomass accumulation is related to protein synthesis. The current study revealed that compared to control N conditions, protein synthesis was promoted by low N conditions and inhibited by high N conditions. Indeed, previous studies have suggested that protein synthesis is one of the main targets for growth control in response to changes in the environment (Wang et al. 2014; Zhou et al. 2017). These results suggest that, differentially abundant proteins involved in cell differentiation, lignin synthesis, and nascent protein synthesis in roots are important factors leading to root morphological changes among the three nitrogen treatments.

## Conclusion

**Fig. 11:**
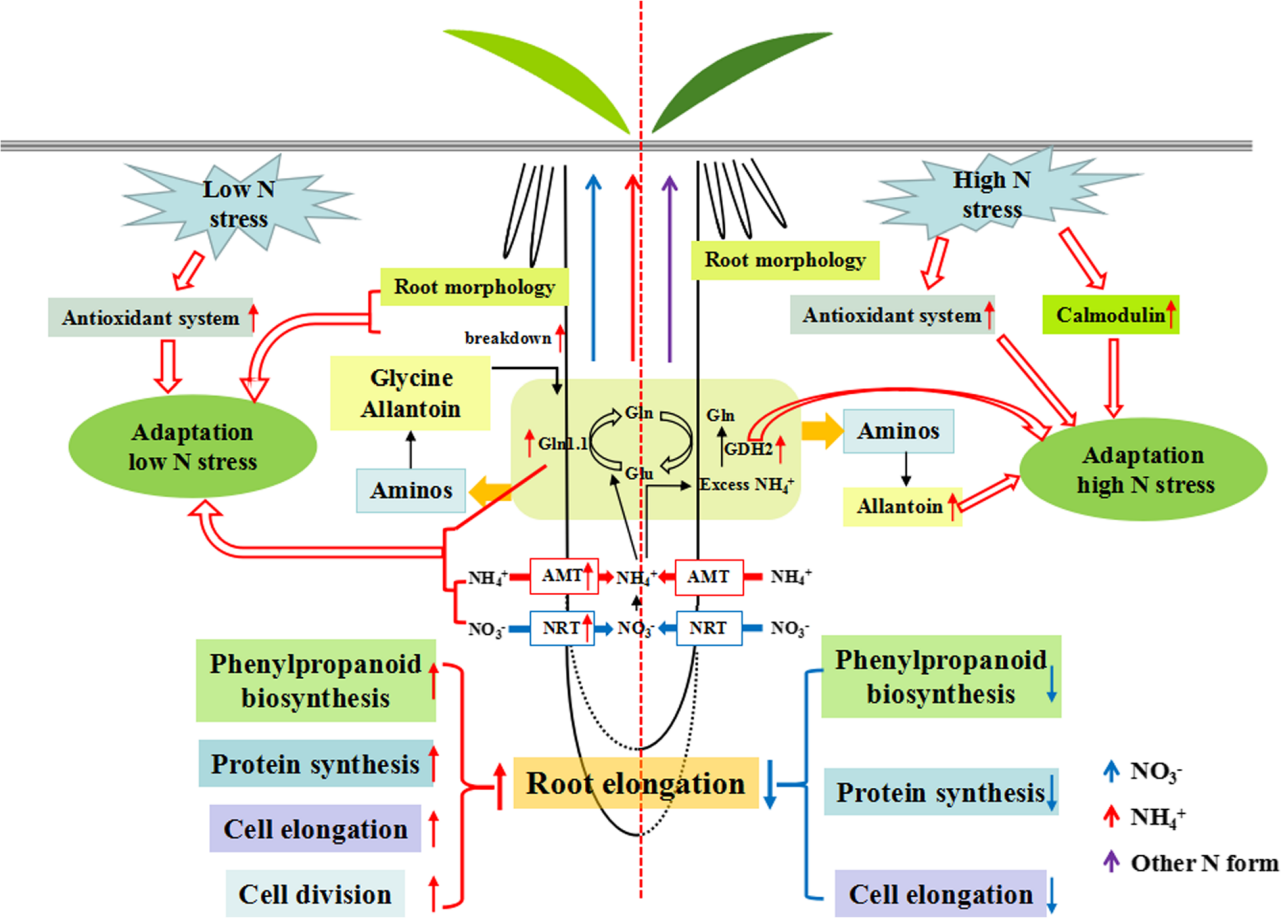
Schematic model representing the growth and stress in the roots of rice when exposed to low and high N conditions

In this study, we compared root morphological and proteomic profiles across three environmental conditions (N levels). Research results suggest that nitrogen may affect root growth by regulating biological processes such as cell development, phenylpropanoid biosynthesis and protein synthesis. In addition, we also found that the adaptation mechanism of rice roots differs greatly under low N and high N conditions. Under low N conditions, rice roots enhance nutrient uptake and utilization by increasing N transport, assimilation and remobilization (glycine, allantoin and protein breakdown) to maintain optimum rice growth and development. Under high N conditions, rice roots determine the abundance of the antioxidant system, calmodulin, allantoin and glutamate dehydrogenase to improve tolerance to high N stress. This study elucidated the molecular mechanism underlying the N-mediated determinants of root growth and development, and provides new insights into improving nitrogen use efficiency in rice (Fig. 11).

## Materials and Methods

### Plant Materials, Mitrogen Treatment and Analysis of Root Characteristics

The experiment was carried out at Shenyang Agricultural University Rice Research Institute, Liaoning Province, China (41°49 N, 123°34 E). Mature rice seeds (cv. ‘shengnong265’ *Japonica* China) were obtained from the Shenyang Agricultural University Rice Research Institute. Seeds were surface-sterilized with 0.01% HgCl_2_, allowed to germinate at 28 °C for 2 d, and further cultured in a greenhouse (average temperature of 28/25 °C, photoperiod of 10 h day/14 h night). At three-leaf stage, the seedlings were subjected to 13.33 ppm (low N), 40 ppm (normal N), or 120 ppm (high N) of nitrogen using NH_4_NO_3_ as the N source and grown in hydroponics for 30 d during the growing season. The formula for the hydroponic solution was slightly improved as described by Li et al. (2012). All treatments had ten replicates with a completely random design. After 30 d (Tillering stage), the relevant indicators were determined.

Roots were sampled after 30 d of treatments, three biological replicates per treatment. Root number, total root length and root diameter were measured with WinRHIZO Pro 2013e software (Regent Instruments Inc., Quebec, Canada). Maximum root length (the length of the longest whole root from root cap to root tip) was measured with a ruler. Then, the roots were dried in an oven at 80 °C to a constant weight for measuring root biomass. Samples were powdered with a micropulverizer (FZ102, Tianjin, China) to measure root nitrogen content using an elemental analyzer (Elementar Vario MACRO cube, Hanau, Germany). Lignin content was determined with the acetyl bromide method as described previously (Foster et al. 2010). Lignin accumulation amount was calculated by lignin content and root biomass.

After 30 d of nitrogen treatment, cell size and number were investigated in 1-cm root segments starting from the root tip. The root segments (three individual rice plants for each treatment) were fixed using 70% FAA fixative. Sectioning and staining were performed using modified method of Qin et al. (2019). Twenty cells were selected from each root segment in the elongation and maturation zone to determine the length and width using CaseViewer (3DHISTECH, Budapest, Hungary). Three fields of each root segment in the meristematic area were selected and measured cell number, record the number of cells in 10,000 μm^2^ (Fig. S2).

For experimental variables, one-way of variance (ANOVA) was applied to assess differences among treatments with SPSS 19.0 (Softonic International, Barcelona, Spain) software and drawn using GraphPad Prism 8 (GraphPad, San Diego, USA) software. Significant differences (*p* < 0.05) between treatments are indicated by different letters according to Fisher’s LSD. The data presented are the means ± standard errors.

### Protein Extraction, Trypsin Digestion and TMT Labeling

Thirty days after nitrogen treatment started, root system samples were collected from 9:00 am to 10:00 am. Three biological replicates per treatment (low N, normal N, and high N) were used for subsequent TMT-based proteomic analysis. Total proteins were extracted using the cold acetone method (Zhang et al. 2018). Samples were ground to a powder in liquid nitrogen and then dissolved in 2 cm^3^ of lysis buffer (8 M urea, 2% SDS, 1× protease inhibitor cocktail (Roche Ltd., Basel, Switzerland)), followed by sonication on ice for 30 min and centrifugation at 13000 g for 30 min at 4 °C. The supernatant was transferred to a fresh tube. For each sample, proteins were precipitated with ice-cold acetone at − 20 °C overnight. The precipitates were washed with acetone three times and redissolved in 8 M urea by sonication on ice. Protein quality was examined by SDS-PAGE. The BCA Protein Assay Kit was used to determine the protein concentration in the supernatant. For each condition, one hundred micrograms of protein was transferred into a new tube and adjusted to a final volume of 100 mm^3^ with 8 M urea. Then, 11 mm^3^ of 1 M DTT (DL-dithiothreitol) was added, and the samples were incubated at 37 °C for 1 h. Then, the samples were centrifuged at 14000 g for 10 min in a 10 K ultrafiltration tube (Millipore). A total of 120 mm^3^ of 55 mM iodoacetamide was added to the sample, and the mixture was incubated for 20 min protected from light at room temperature. The precipitates were washed by 1 cm^3^ pre-chilled 90% acetone aqueous solution for twice and then re-dissolved in 100 mm^3^ 100 mM TEAB. The proteins were then digested with sequence-grade modified trypsin (Promega, Madison, WI) at 37 °C overnight. Then, the digested samples were centrifuged at 13500 g for 12 min, dried under vacuum, and dissolved in 500 mM TEAB. The resultant peptide mixture was labeled with TMT- 10Plex Isobaric Mass Tag Labeling Kit (Thermo Fisher Scientific, MA, USA) for 2 h at room temperature. The labeled peptide samples were then pooled and lyophilized in a vacuum concentrator.

### HPLC Fractionation and LC-MS/MS Analysis

The peptide mixture was redissolved in buffer A (buffer A: 20 mM ammonium formate in water, pH 10.0, adjusted with ammonium hydroxide) and then fractionated by high-pH separation using an Ultimate 3000 system (Thermo Fisher Scientific, MA, USA) connected to a reversed-phase column (XBridge C18 column, 4.6 mm × 250 mm, 5 μm, Waters Corporation, MA, USA). High-pH separation was performed using a linear gradient starting from 5% B to 45% B over 40 min (buffer B: 20 mM ammonium formate in 80% ACN, pH 10.0, adjusted with ammonium hydroxide). The column was re-equilibrated to the initial conditions for 15 min. The column flow rate was maintained at 1 cm^3^/min, and the column temperature was maintained at 30 °C. Twelve fractions were collected; each fraction was dried in a vacuum concentrator for the next step.

Peptide fractions were resuspended with 30 mm^3^ of solvent C (C: water with 0.1% formic acid; D: ACN with 0.1% formic acid), separated by nanoLC and analyzed by online electrospray tandem MS. The experiments were performed on an Easy-nLC 1000 system (Thermo Fisher Scientific, MA, USA) connected to an Orbitrap Fusion Tribrid mass spectrometer (Thermo Fisher Scientific, MA, USA) equipped with an online nanoelectrospray ion source. A 10-mm^3^ peptide sample was loaded onto the trap column (Thermo Scientific Acclaim PepMap C18, 100 μm × 2 cm) with a flow of 10 mm^3^/min for 3 min and subsequently separated on an analytical column (Acclaim PepMap C18, 75 μm × 15 cm) with a linear gradient from 2% D to 40% D in 70 min. The column was re-equilibrated to the initial conditions for 10 min. The column flow rate was maintained at 300 μm^3^/min. An electrospray voltage of 2 kV against the inlet of the mass spectrometer was used. The Fusion mass spectrometer was operated in data-dependent acquisition mode to switch automatically between MS and MS/MS acquisition. Full-scan MS spectra (m/z 350–1550) were acquired with a mass resolution of 120 K, followed by sequential high-energy collisional dissociation (HCD) MS/MS scans with a resolution of 30 K. The intense signals in the MS spectra (> 1e4) were subjected to an additional MS/MS analysis. The automatic gain control (AGC) values for MS and MS/MS were set to 4e5 and 8e4, respectively. The maximum ion injection times for MS and MS/MS were 50 and 100 ms, respectively. The isolation window was set to 1.6 Da. In all cases, one microscan was recorded using dynamic exclusion of 30 s.

### Database Search

Tandem mass spectra were extracted, charge state deconvoluted and deisotoped by Mascot Distiller version 2.6. Then, the MS data were transformed into MGF files with Proteome Discoverer 1.2 (Thermo, Pittsburgh, PA, USA) and analyzed using the Mascot search engine (Matrix Science, London, UK; version 2.3.2). The Mascot database was set up for protein identification using the rice (*Oryza sativa*) protein information from IRGSP-1.0 in ensemble (version 38) database with the following parameters: digestion enzyme, trypsin; 1 missed cleavage allowed. Mascot was searched with a fragment ion mass tolerance of 0.050 Da and a parent ion tolerance of 20.0 ppm. Carbamidomethyl on cysteine and TMT-10-plex on lysine and the N-terminus were specified in Mascot as fixed modifications. Deamidation of asparagine and glutamine, and oxidation of methionine and acetyl at the N-terminus were specified in Mascot as variable modifications.

### Protein Identification, Quantification and Annotation Analysis

Protein identification results were accepted if a false discovery rate (FDR) of less than 1.0% was achieved by the Scaffold Local FDR algorithm. Proteins that contained similar peptides and could not be differentiated based on MS/MS analysis alone were grouped to satisfy the principles of parsimony. Protein quantification was carried out for those proteins identified in all the samples with ≥2 unique spectra. Protein relative quantification was based on the ratios of reporter ions, which reflect the relative abundance of peptides. The Mascot search results were averaged using medians and quantified. Fold changes > 1.2 and t-test *p* < 0.05 were used as a threshold to identify the significantly different proteins. Proteins were annotated against the GO (http://www.gene-ontology.org) and KEGG (http://kobas.cbi.pku.edu.cn/) databases for identification of function. Significant GO functions and pathways were examined within DAPs with *p* value ≤0.05. Heatmap analysis was performed using the OmicShare tools, a free online platform for data analysis (http://www.omicshare.com/tools).

### Total RNA Extraction and Verification of the Proteomic Profile

Total RNA extraction was performed with the Eastep Super Total RNA Extraction Kit (Promega), and first-strand cDNA was synthesized with the PrimeScript RT Master Mix (TaKaRa). Real-time RT-PCR was conducted based on the method described by Xin et al. (2019a). The primers used for RT-PCR analysis are listed in Table S3. Calculation of correlation coefficients and plotting of the proteomic profile and RT-PCR data were performed with Origin 9 (Northampton, MA, USA) software.

## Supplementary Information

**Additional file 1: Figure S1.** Basic protein identification information statistics by TMT analysis.

**Additional file 2: Figure S2.** Schematic diagram of meristem, elongation and maturity zone of the root tip.

**Additional file 3: Table S1**. Abbreviations list.

**Additional file 4: Table S2.** Proteomic experiment design.

**Additional file 5: Table S3**. Sequences of primers used in this study.

**Additional file 6: Table S4**. DAPs information involved in nitrogen metabolism, cell structure and growth, protein homeostasis, and stress related proteins under low and high N conditions.

**Additional file 7: Table S5**. Peptide information list for protein identification results.

**Additional file 8: Table S6**. Protein information list for protein identification results.

## Data Availability

The mass spectrometry proteomics data have been deposited to the ProteomeXchange Consortium (http://proteomecentral.proteomexchange.org) via the PRIDE partner repository with the dataset identifier PXD016653. After the data have been published, the access connection in ProteomeXchange will be: http://proteomecentral.proteomexchange.org/cgi/GetDataset? ID = PXD016653.

## Abbreviations

AAH: Allantoate deiminase
AGD12.1: ADP-ribosylation factor GTPase-activating protein
AGD12.2: ADP-ribosylation factor GTPase-activating protein
AGT2: Alanine-glyoxylate aminotransferase 2
ALAAT: Alanine aminotransferase 2
ALN: Allantoinase
AMAT: Methanol O-anthraniloyltransferase
AMP1: Glutamate 2
AMT1.3: Ammonium transporter 1 member 3
ANNAT7: Annexin D7
AOX1A: Ubiquinol oxidase
APX1: L-ascorbate peroxidase 1
APX2: L-ascorbate peroxidase 2
ARD2: Acireductone dioxygenase 2
ARP5: Actin-related protein 5
ASB1: Anthranilate synthase beta subunit 1
ASN: Asparagine synthetase 1
ASP2: Aspartic proteinase Asp1-like
ASPG1.1: ASPARTIC PROTEASE IN GUARD CELL 1.1
ASPG1.2: ASPARTIC PROTEASE IN GUARD CELL 1.2
ATJ15: Chaperone protein dnaJ 15
BCDH BETA1: 2-oxoisovalerate dehydrogenase subunit beta 1
BGLU13: Beta-glucosidase 13
BGLU4: Beta-glucosidase 4
CAM 1.1: Calmodulin-1.1
CAM 1.3: Calmodulin-1.3
CAM2: Calmodulin-2
CAM7: Calmodulin-7
CATB: Catalase isozyme B
CCP1: Cysteine proteinase 1 precursor
CDC5: Cell division cycle 5
CGS1: Cystathionine gamma-synthase 1
CML16: Calcium-binding protein CML16
COX5B-2: Cytochrome c oxidase subunit 5b-1
CPR: NADPH--cytochrome P450 reductase
CXP2.2: Serine carboxypeptidase II-2
CXP2.3: Serine carboxypeptidase II-3
CYP84A1: Cytochrome P450 84A1
CYP93G1: Cytochrome P450 93G1
DAO2: Amine oxidase
DAPC: N-succinyldiaminopimelate aminotransferase
DAPs: Differentially abundant proteins
DHAD: Dihydroxy-acid dehydratase
DMAS1: NAD(P)H-dependent oxidoreductase 1
EDA2: Serine protease
EF1: Elongation factor 1-delta 1
EIF-4G: Eukaryotic translation initiation factor 4G
ELC: Protein ELC
ERVB: Ervatamin-B
EXPA8: Expansin-A8
FAD: Inactive tetrahydrocannabinolic acid synthase
FIS1: 1-pyrroline-5-carboxylate dehydrogenase
GDCSP: Glycine dehydrogenase
GDH2: Glutamate dehydrogenase 2
GEK1: D-aminoacyl-tRNA deacylase
GGT3: Glutathione gamma-glutamylcysteinyltransferase
GLN1.1: Glutamine synthetase cytosolic isozyme 1–1
GLN1.2: Glutamine synthetase cytosolic isozyme 1–2
GLT1: Glutamate synthase 2
GOGA5: Golgin-84
GOT: Aspartate aminotransferase
GOT2: Aspartate aminotransferase 2
GPT: L-alanine:2-oxoglutarate aminotransferase
GRPE: GrpE nucleotide exchange factor domain-containing protein
GRXS1: Monothiol glutaredoxin-S1
GSTU 44: Glutathione S-transferase 44
GSTU 48: Glutathione S-transferase 48
GSTU 50: Glutathione S-transferase 50
GSTU1: Glutathione S-transferase 1
GSTZ1: Glutathione S-transferase 2
GXM2: Glucuronoxylan 4-O-methyltransferase 1
High N: High nitrogen
HSP60.2: Chaperonin CPN60.2
ICLN: Chloride conductance regulatory
ISPH: 4-hydroxy-3-methylbut-2-enyl diphosphate reductase
KINESIN-13A: Kinesin-13A
LHPI: Ornithine cyclodeaminase
Low N: Low nitrogen
LPR1: Multicopper oxidase
MAP 3 K: PI-PLC X domain-containing protein
MJ0531: Universal stress protein MT2085 isoform X2
N: Nitrogen
NAAT-A: Nicotianamine aminotransferase A
NEP1: Aspartic proteinase nepenthesin-1
Normal N: Normal nitrogen
NPD: Endoglucanase
NPF2.11: Protein NRT1/ PTR FAMILY 2.11
NRT2.3: High-affinity nitrate transporter 2.3
NUP98A: Nuclear pore complex protein
OCP: Oryzain alpha chain precursor
OMT2: O-methyltransferase 2
OPR4: Putative 12-oxophytodienoate reductase 4
P5CS: Delta-1-pyrroline-5-carboxylate synthase
PATL5.1: Patellin-3.1
PATL5.2: Patellin-3.2
PDX11: Pyridoxal 5′-phosphate synthase subunit
PER1: Peroxidase 1
PER12: Peroxidase 12
PER72: Peroxidase 72
PLP3B: Thioredoxin domain-containing
PME3: Pectinesterase
POD: Peroxidase A2
POMGNT2: Protein O-linked-mannose beta-1,4-N-acetylglucosaminyltransferase 2
PRS13.2: 40S ribosomal protein S13.2
PRX 95: Peroxidase 95
PRX 98: Peroxidase 98
PRX74: Peroxidase 74
PSMC6: 26S protease regulatory subunit 6B homolog
PUX4: Plant UBX domain-containing protein 4
QOR: Oxidoreductase
RIC1: Ras-related protein RIC1
RKL1: Leucine Rich Repeat family protein
RPL15: 60S ribosomal protein L15
RPL15.1: 60S ribosomal protein L15.1
RPL27C: 60S ribosomal protein L27–3
RPL35AB: 60S ribosomal protein L35a-3
RPL35AC: 60S ribosomal protein L35a-1
RPL37a: 60S ribosomal protein L37a isoform 2
RPL37b: 60S ribosomal protein L37a
RPL4a: 60S ribosomal protein L4a
RPL4b: 60S ribosomal protein L4b
RPL6: 60S ribosomal protein L6–3
RPL7a: 60S ribosomal protein L7a
RPL7b: 60S ribosomal protein L7b
RPN10: 26S proteasome non-ATPase regulatory subunit 4
RPP2A: 60S acidic ribosomal protein P2A
RPP2B: 60S acidic ribosomal protein p2b
RPS11.1: 40S ribosomal protein S11.1
RPS11.2: 40S ribosomal protein S11.2
RPS11.3: 40S ribosomal protein S11.3
RPS16a: 40S ribosomal protein S16a
RPS16b: 40S ribosomal protein S16b
RPS24B: 40S ribosomal protein S24–1
RPS28: Os10g0411700, partial
RPS2D: 40S ribosomal protein S2–3
RPS9b: 40S ribosomal protein S9b
RPS9c: 40S ribosomal protein S9c
RPS9d: 40S ribosomal protein S9d
SBT1.7: Subtilisin-like protease SBT1.7
SBT3.5: Subtilisin-like protease SBT3.5
SC34: 60S ribosomal protein L10–1
SCP41: Peptidase S10
SCPL50.1: Serine carboxypeptidase-like 50
SCPL50.2: Serine carboxypeptidase 50
SCPL51: Serine carboxypeptidase-like 51
SEN102: Zingipain-2-like
SPN1: Serpin-ZXA
SYP132: Syntaxin-132
TAX10: 3′-N-debenzoyl-2′-deoxyTaxol N-benzoyltransferase
TBCLD15: TBC1 domain family member 15
TIM9: Mitochondrial import inner membrane translocase subunit
TON1B: Protein TONNEAU 1b
TON1C: Protein TONNEAU 1c
TOR1: Microtubule-associated protein SPIRAL2
TR1: Tropinone reductase
TRXH: Thioredoxin H1
TTL: Uric acid degradation bifunctional protein
TUBA1: Tubulin alpha-1
TUBB1: Tubulin beta-1
TUBB3: Tubulin beta-3
TUBB5: Tubulin beta-5
TUBB6: Tubulin beta-6
UOX: Uricase
VPE2: Vacuolar-processing enzyme
VPS2.1: Vacuolar protein sorting-associated protein 2.1
WWOX: Retinol dehydrogenase 12
XCP2: Xylem cysteine proteinase 2
XTH22: Xyloglucan endotransglucosylase 22
XTH8: Xyloglucan endotransglycosylase 8
YKT61: VAMP-like protein YKT61
