# Supplementary figures and images for: Adaptation Mechanism of Roots to Low and High Nitrogen Revealed by Proteomic Analysis
Source: Rice (N Y). 2021 Jan 7;14:5. doi: 10.1186/s12284-020-00443-y (PMC7790981; doi:10.1186/s12284-020-00443-y)

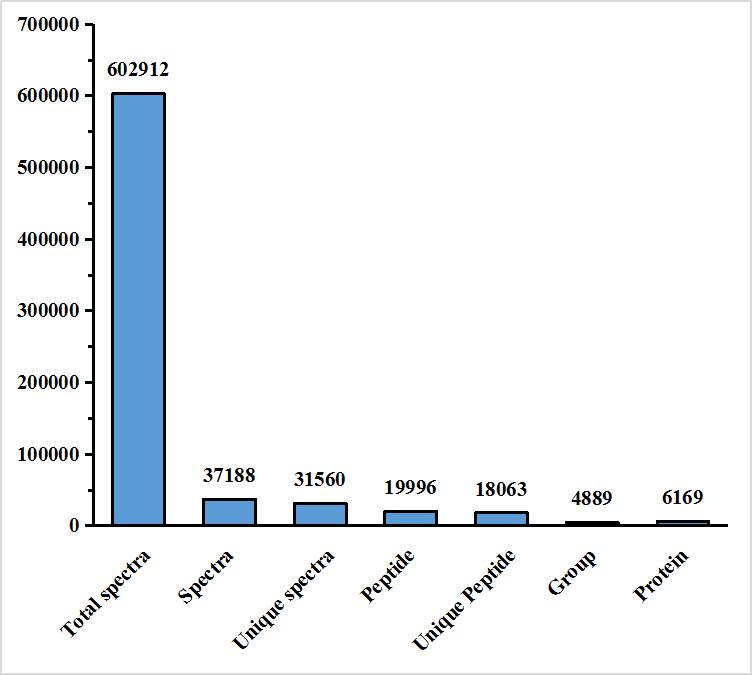


**Supplementary Figure 1.** Basic protein identification information statistics by TMT analysis

Supplement: Supplementary file 1 — Additional file 1: Figure S1. Basic protein identification information statistics by TMT analysis. [file 12284_2020_443_MOESM1_ESM.docx]

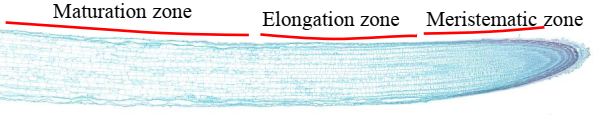
 **Supplementary Figure 2.** Schematic diagram of meristem, elongation and maturity of the root tip.

Supplement: Supplementary file 2 — Additional file 2: Figure S2. Schematic diagram of meristem, elongation and maturity zone of the root tip. [file 12284_2020_443_MOESM2_ESM.docx]
